# Spadin Selectively Antagonizes Arachidonic Acid Activation of TREK-1 Channels

**DOI:** 10.3389/fphar.2020.00434

**Published:** 2020-04-07

**Authors:** Ruolin Ma, Anthony Lewis

**Affiliations:** School of Pharmacy and Biomedical Sciences, Institute of Biomedical and Biomolecular Sciences, University of Portsmouth, Portsmouth, United Kingdom

**Keywords:** spadin, TREK-1, TREK-2, antagonism, arachidonic acid

## Abstract

TREK-1 channel activity is a critical regulator of neuronal, cardiac, and smooth muscle physiology and pathology. The antidepressant peptide, spadin, has been proposed to be a TREK-1-specific blocker. Here we sought to examine the mechanism of action underlying spadin inhibition of TREK-1 channels. Heterologous expression in *Xenopus laevis* oocytes and electrophysiological analysis using two-electrode voltage clamp in standard bath solutions was used to characterize the pharmacological profile of wild-type and mutant murine TREK-1 and TREK-2 channels using previously established human K_2P_ activators; arachidonic acid (AA), cis-4,7,10,13,16,19-docosahexaenoic acid (DHA), BL-1249, and cinnamyl-3,4-dihydroxy-α-cyanocinnamate (CDC) and inhibitors; spadin and barium (Ba^2+^). Mouse TREK-1 and TREK-2 channel currents were both significantly increased by AA, BL-1249, and CDC, similar to their human homologs. Under basal conditions, both TREK-1 and TREK-2 currents were insensitive to application of spadin, but could be blocked by Ba^2+^. Spadin did not significantly inhibit either TREK-1 or TREK-2 currents either chemically activated by AA, BL-1249, or CDC, or structurally activated *via* a gating mutation. However, pre-exposure to spadin significantly perturbed the subsequent activation of TREK-1 currents by AA, but not TREK-2. Furthermore, spadin was unable to prevent activation of TREK-1 by BL-1249, CDC, or the related bioactive lipid, DHA. Spadin specifically antagonizes the activation of TREK-1 channels by AA, likely *via* an allosteric mechanism. Lack of intrinsic activity may explain the absence of clinical side effects during antidepressant therapy.

## Introduction

Two-pore domain (K_2P_) channels generate a background or leak potassium (K^+^) ion conductance critical to the control of cell membrane potential and excitability (Goldstein et al., 2001; Feliciangeli et al., 2015). Fifteen mammalian genes (KCNK) encode K_2P_ subunits containing four transmembrane domains (TM1-4) flanking two pore-forming domains (P1, P2), that associate *via* homo or heteromeric dimerization to generate functional membrane proteins (Renigunta et al., 2015). TWIK-related K^+^ channels (TREK-1, TREK-2, and TRAAK) are a mechano-gated subclass of K_2P_ channels, and are highly expressed throughout the nervous system (Talley et al., 2001) as well as in several non-neuronal tissues including cardiac and smooth muscles (Gurney and Manoury, 2009; Schmidt et al., 2014; Ma et al., 2018). TREK-1 in particular plays a central role in pain perception, neuroprotection, and cardiac rhythmogenesis (Alloui et al., 2006; Wiedmann et al., 2016; Lamas and Fernandez-Fernandez, 2019) and is considered a viable therapeutic target for treating depression (Heurteaux et al., 2006), atrial fibrillation (Lugenbiel et al., 2017), and hypermotility disorders of the gastrointestinal tract (Ma et al., 2018).

Gating of TREK channels is polymodal, and open probability can be determined by a plethora of physiochemical signals including pH, mechanical stretch, temperature, and bioactive lipids (Noel et al., 2011). Specifically, TREK-1 and TREK-2 channels can be strongly and reversibly activated by polyunsaturated free fatty acids (PUFAs) such as AA and DHA through a mechanism involving the C-terminal domain (Fink et al., 1998; Patel et al., 1998; Maingret et al., 1999; Ferroni et al., 2003). Other activators including caffeic acid esters [such as cinnamyl-3,4-dihydroxy-α-cyanocinnamate (CDC)] and cyclooxygenase inhibitors including BL-1249 and FFA (Danthi et al., 2004; Veale et al., 2014) work independently of the C-terminal domain and are thought to bind below the selectivity filter to open the filter gate (Schewe et al., 2019). Conversely, there is a surprising paucity of TREK channel blockers that have the potential to be exploited for therapeutic purposes. TREK channels are not sensitive to classical potassium channel blockers like TEA, 4-AP, and cesium ions (Cs^+^) (Fink et al., 1996; Koh et al., 2001; Cadaveira-Mosquera et al., 2011), but can be inhibited by barium ions (Ba^2+^) with an IC_50_ ~1 mM (Ma et al., 2011). TREK-1 channels are also sensitive to selective serotonin reuptake inhibitors (SSRIs) such as fluoxetine (Kennard et al., 2005) which are thought to bind within intramembrane fenestrations in a state-dependent manner (Dong et al., 2015; Mcclenaghan et al., 2016).

Recently a TREK-1 specific inhibitor with therapeutic potential to treat depression was identified (Mazella et al., 2010; Moha Ou Maati et al., 2012). Spadin is an engineered fragment of a natural NTSR3/sortilin propeptide released into blood after the cleavage of prosortilin by the protein convertase, furin. Spadin binds with high affinity to the neurotensin (NT) receptor which has been shown to physically associate with the TREK-1 channel, regulating its cell surface expression (Mazella et al., 2010). Spadin has also been shown biochemically to bind to TREK-1, and suggested to inhibit channel currents but only when pre-activated with AA (Mazella et al., 2010), indicating state-dependent association. Here, using murine forms of TREK-1 and TREK-2 heterologously expressed in *Xenopus* oocytes, we sought to understand the mechanism of spadin inhibition of TREK-1 channel currents.

## Materials and Methods

### Molecular Biology

Mouse TREK-1 (mTREK-1) and mouse TREK-2 (mTREK-2) were a kind gift from Guillaume Sandoz (Université de Nice Sophia Antipolis), while human TREK-1 (hTREK-1) was kindly provided by Dierk Thomas (University of Heidelberg). Plasmid DNA was isolated using the Qiagen Miniprep Kit (Qiagen, Valencia, CA), and all constructs were sequenced by Eurofins Genomics (Ebersberg, Germany). All plasmid DNA were sub-cloned into our in-house pMAX expression vector (based on pcDNA3.1), linearized with PacI (New England Biolabs), and purified using a QIAquick PCR Purification Kit (Qiagen, Valencia, CA). Site-directed mutagenesis was performed by PCR. cRNA transcripts were generated by *in-vitro* transcription using the T7 mMESSAGE mMACHINE kit (Ambion, Inc., Austin, TX). cRNA concentration and purity was determined by spectrophotometry and visualized for qualitative analysis by gel electrophoresis.

### Oocyte Preparation and RNA Injection

*Xenopus laevis* ovaries were sourced from the European Xenopus Resource Centre (University of Portsmouth, Portsmouth, UK). Experiments were approved by the Animal Welfare and Ethics Review Committee of the University of Portsmouth, and were performed in accordance with the Animals (Scientific Procedures) Act 1986 (UK). Stage IV–V oocytes were isolated and defolliculated in Ca^2+^-free OR-2 solution (in mM: 82.5 NaCl, 2.5 KCl, 1 MgCl_2_, 10 HEPES, pH 7.4 tris-base) using a combination of manual dissection and collagenase treatment (~1 h in 2 mg/ml Worthington type II collagenase, Lorne Labs, UK). Oocytes were injected with cRNA (2–10 ng) and incubated at 16°C in ND-96 solution (in mM: 96 NaCl, 2 KCl, 1 MgCl_2_, 1.8 CaCl_2_, 10 HEPES, pH 7.6, tris-base) containing 50 µg/ml gentamycin and 1% penicillin/streptomycin, for at least 24 h before analysis.

### Electrophysiology

Whole-cell currents in oocytes were recorded 1–3 days post-injection by two-electrode voltage clamp (TEVC) using a Warner TEV700 oocyte clamp amplifier (Harvard Apparatus, UK) and Axon Digidata 1440A (Molecular Devices, UK). Microelectrodes were back filled with 3 M KCl and had tip resistances of 0.2–1.0 MΩ. Currents were recorded at room temperature (21 ± 1°C) with constant solution flow at 1–2 ml/min with bath solution containing (in mM): 96 NaCl, 4 KCl, 1 MgCl_2_, 0.3 CaCl_2_, 10 HEPES (pH 7.6, tris-base). Data were sampled at 5 kHz, filtered at 1 kHz and recorded using Clampex 10.1 software (Axon Instruments, Inc.). Currents were not adjusted for leak or capacitance. Oocyte membrane potential was held at −80 mV and step depolarized to potentials ranging from −120 mV to +60 mV in 10 mV steps for a duration of 500 ms.

### Data and Statistical Analysis

The raw data supporting the conclusions of this manuscript will be made available by the authors, without undue reservation, to any qualified researcher. Data was analyzed using Clampfit 10.7 (Axon Instruments, Inc.) and tabulated in Excel (Microsoft Office 2013). Current-voltage (I-V) relationships were generated from steady-state currents at potentials between −120 mV and +60 mV. Statistical analyses and graphical evaluations were performed with Prism 7.0 (GraphPad, Inc., La Jolla, CA, USA). For all analysis, *n* values represent oocytes numbers. All data are presented as the arithmetic mean ± SEM unless stated otherwise. Statistical comparisons were made using either Student's paired *t* test or one-way ANOVA (not repeated measures). A *p* < 0.05 was considered statistically significant.

### Drugs

Arachidonic acid (AA), BL-1249, CDC, cis-4,7,10,13,16,19-docosahexaenoic acid (DHA), and barium chloride (BaCl_2_) were purchased from Sigma-Aldrich, spadin was bought from Tocris. All substances, except BaCl_2_, were stored as stocks (1 mM–1 M) at −20°C and diluted to the required concentrations in standard bath solution immediately prior to experimentation.

## Results

### Mouse TREK-1 and TREK-2 Channels Are Biophysically and Pharmacologically Comparable to Their Human Counterparts

**Figure 1:**
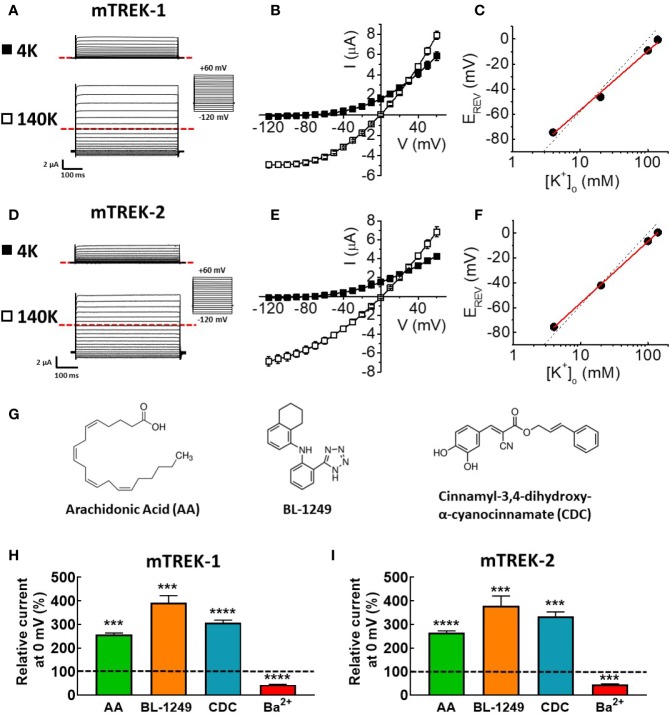
Mouse TREK-1 and TREK-2 channels are biophysically and pharmacologically comparable to their human counterparts. Exemplar current traces of mTREK-1 **(A)** and mTREK-2 **(D)** elicited by voltage pulses from −120 to +60 mV in 10 mV steps, for 500 ms from a holding potential of −80 mV (inset), in both asymmetric K^+^ solutions (4 mM) or symmetric K^+^ solutions (140 mM). In each panel, scale bars represent 2 μA and 100 ms. The zero current level is indicated by red dashed line. **(B, E)** Mean current-voltage (I-V) relationships taken from steady-state currents in bath solution containing low (asymmetric) K^+^ (4 mM, black squares) or high (symmetric) K^+^ (140 mM, open squares) for **(B)** mTREK-1 (*n* = 7) and **(E)** mTREK-2 (*n* = 8). **(C, F)** Reversal potential (E_REV_) plotted as a function of [K^+^]_o_ (mean ± SEM) for **(C)** mTREK-1 and **(F)** mTREK-2 currents. Data points are fitted with a linear relationship (red line). Dashed line represents Nernstian theoretical linear relationship of equilibrium potential for K^+^ (E_K_) with changing [K^+^]_o_ as calculated using the Nernst equation, assuming [K^+^]_i_ of 140 mM (slope = −58.2 mV). **(G)** Chemical structures of three main activators of TREK channels. **(H, I)** Bar graphs showing effects on mean (± SEM) normalized mTREK-1 **(H)** and mTREK-2 **(I)** currents measured at 0 mV (in %) after application of either 10 μM AA, 1 μM BL-1249 (mTREK-1), 3 μM BL-1249 (mTREK-2), 10 μM CDC, or 1 mM Ba^2+^. Dashed line indicates control current level. All data represents *n* > 6 oocytes. ***p < 0.001, ****p < 0.0001, Student's paired *t*-test conducted on raw data.

In order to allow a direct comparison to previous work by ourselves and others using mouse model tissue, we conducted a comprehensive biophysical and pharmacological assessment of cloned mouse TREK-1 and TREK-2 channels by two-electrode voltage clamp after heterologous expression in *X. laevis* oocytes. Robust ionic currents were recorded in standard bath solution containing 4 mM K^+^ from both mTREK-1 (**Figure 1A**) and mTREK-2 (**Figure 1D**) expressing oocytes. Such significant currents were absent in water-injected oocytes. The phenotype of the two channel currents resembled that of their well-characterized human K_2P_ channel counterparts; *quasi* time-independent activation and strong outwardly rectifying currents in physiological solutions with minimal inward currents observed in 4 mM K^+^ (**Figures 1B, E**).

When external K^+^ concentration ([K^+^]_o_) was increased to 140 mM K^+^, prominent inward currents were revealed for both channels at voltages below the predicted equilibrium potential (E_K_ = 0 mV, **Figures 1B, E**). Incremental increases in extracellular K^+^ concentration from 4 mM to 140 mM induced a parallel shift in the voltage threshold at which outward ionic currents were observed (E_REV_). A plot of E_REV_ as a function of [K^+^]_o_ revealed slopes of 47.3 ± 2.2 mV (**Figure 1C**, mTREK-1) and 49.4 ± 0.4 mV (**Figure 1F**, mTREK-2) close to the calculated Nernst value of 58.2 mV indicating these two channels are highly selective for K^+^ ions.

Previous studies have established that human TREK channel currents are sensitive to a wide range of pharmacological agents, including activation by structurally diverse compounds such as AA (Fink et al., 1998; Patel et al., 1998; Lesage et al., 2000), BL-1249 (Veale et al., 2014), and CDC (Danthi et al., 2004), the structures of which are shown in **Figure 1G**.

Analysis of mTREK-1 currents at recorded at 0 mV revealed a significant increase in current magnitude upon application of 10 μM AA (256.8 ± 6.7%), 1 μM BL-1249 (392.9 ± 29.5%), and 10 μM CDC (307.8 ± 10.1%) respectively (**Figure 1H**). Application of 1 mM Ba^2+^ caused a significant reduction of mTREK-1 currents to 56.1 ± 1.4% at 0 mV compared to control (**Figure 1H**).

Similar activation and inhibition was seen in mTREK-2 channels. mTREK-2 currents recorded at 0 mV were significantly increased by application of 10 μM AA (265.77 ± 6.5%), 3 μM BL-1249 (333.7 ± 19.3%), and 10 μM CDC (307.8 ± 10.1%) respectively (**Figure 1I**). Similar to mTREK-1, mTREK-2 currents recorded at 0 mV were significantly inhibited in the presence of 1 mM Ba^2+^ to 55.5 ± 3.4% (**Figure 1I**). These results reveal that murine TREK-1 and TREK-2 channels possess a similar pharmacological profile as their human counterparts; activation by AA, BL-1249, and CDC, and inhibition by barium.

### Spadin Specifically Antagonizes AA-Activation of TREK-1 Channels

**Figure 2:**
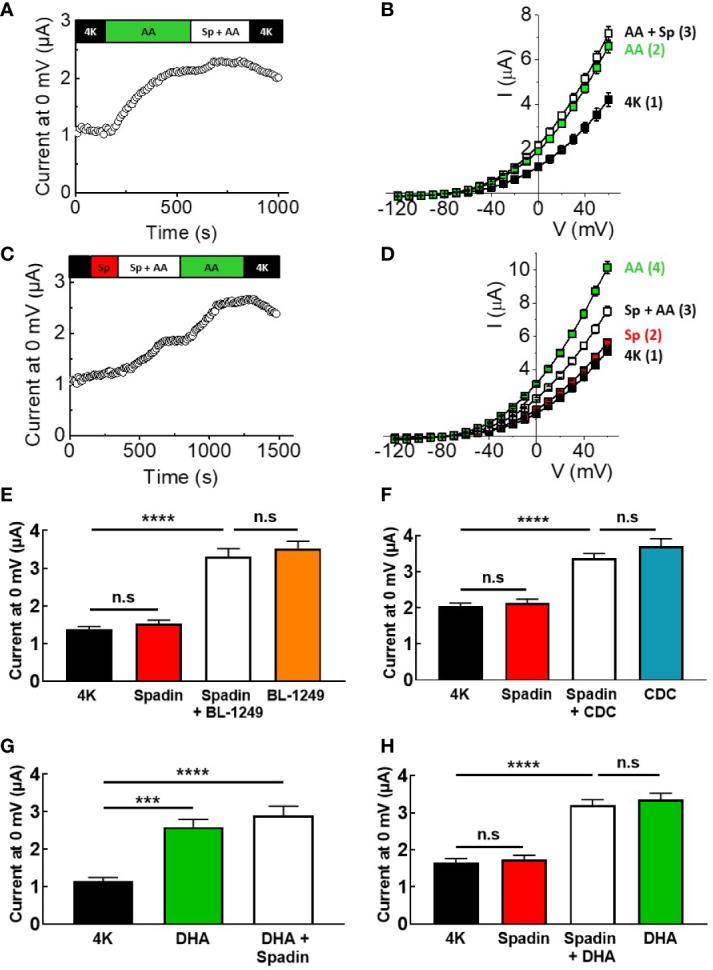
Spadin specifically antagonizes AA-activation of TREK-1 channels. **(A, C)** Exemplar time-courses of mTREK-1 currents measured at 0 mV recorded from oocytes by TEVC, under different bath conditions; control (4K, black), 1 μM spadin alone (Sp, red), 10 μM AA alone (AA, green), or 1 μM spadin plus 10 μM AA (Sp +AA, white) as indicated on bar. **(B, D)** Mean current-voltage relationships of experiments from A and C respectively (*n* = 8–12). Numbers in brackets refer to the order of application of compounds, mirroring respective time-courses. **(E, F)** Mean bar graphs showing the effect of spadin on mTREK-1 current activation by **(E)** BL-1249 and **(F)** CDC. mTREK-1 current amplitudes were recorded at 0 mV by TEVC in control bath conditions (4K, black), and following pre-treatment with 1 μM spadin alone (red), 1 μM spadin plus indicated activator (white) and activators alone; 1 μM BL-1249 (orange) or 10 μM CDC (blue). **(G, H)** Mean bar graphs showing the effect of spadin on mTREK-1 currents amplitudes recorded at 0 mV by TEVC in the presence of DHA. **(G)** Mean mTREK-1 currents recorded in control bath conditions (4K, black), and following pre-activation with 10 μM DHA (green) and when supplemented with 1 μM spadin (white). **(H)** Mean mTREK-1 currents recorded in control bath conditions (4K, black), and following pre-treatment with 1 μM spadin alone (red), supplementation with 10 μM DHA (white) or 10 uM DHA alone (green). Data are presented as mean ± SEM, *n* = 10 for both. *n.s*, not significant (*p* > 0.05), ****p < 0.0001, one-way ANOVA followed by Tukey's multiple comparisons test.

Spadin has previously been suggested to be a specific blocker of TREK-1 channels, acting *via* a direct channel interaction, with an IC_50_ of ~70 nM at 0 mV after pre-activation by AA (Mazella et al., 2010). Here, mTREK-1 channels pre-activated with 10 μM AA were unable to be significantly inhibited by 1 μM spadin (**Figures 2A, B**), a concentration predicted to induce ~75% inhibition (Mazella et al., 2010). At 0 mV, mean AA-activated current mTREK-1 current was 1.9 ± 0.1 μA compared to 2.1 ± 0.1 μA following the addition of spadin. Furthermore, mTREK-1 basal channel activity was also insensitive to spadin, with channel currents at 0 mV measured as 1.4 ± 0.1 μA (control mTREK-1 current) versus 1.5 ± 0.1 μA (1 μM spadin) (**Figures 2C, D**).

Given that spadin did not appear to be a traditional blocker of mTREK-1 channels, we decided to investigate whether spadin could antagonize AA-activation of mTREK-1 channels. Oocytes were incubated for 3–5 min in bath solution containing 1 μM spadin before addition of 10 μM AA (time-course shown in **Figure 2C**). Pre-application of 1 μM spadin significantly perturbed the activation of mTREK-1 channels by AA by 52.2 ± 14.7%, when compared to AA-activated currents after the removal of spadin (**Figures 2C, D**). This was not a species-specific phenomenon. Spadin was also unable to block AA-activated currents generated by human TREK-1, and induced comparable antagonism of AA-activation of hTREK-1 currents (**Supplementary Figure 1**).

In order to begin to understand the mechanism of action of spadin, we next wanted to assess the antagonistic activity of spadin against other known TREK-1 activators. Spadin was unable to prevent BL-1249 (1 μM) stimulation of mTREK-1 currents at 0 mV; 3.3 ± 0.2 μA versus 3.5 ± 0.2 μA in the presence and absence respectively of spadin (**Figure 2E**), Similarly spadin was unable to prevent CDC (10 μM) stimulation of mTREK-1 currents at 0 mV; 3.4 ± 0.1 μA versus 3.7 ± 0.2 μA in the presence and absence respectively of spadin (**Figure 2F**).

DHA is a polyunsaturated fatty acid (PUFA) closely related to AA, known to activate TREK-1 channels (Patel et al., 2001). Application of 10 μM DHA, as expected, exhibited a significant enhancement of mTREK-1 currents, with an increase of 2.3 ± 0.2-fold at 0 mV compared to control (**Figure 2G**). Spadin was unable to block the DHA-activated mTREK-1 currents [**Figure 2G**; 2.6 ± 0.2 μA (DHA activated current) versus 2.9 ± 0.3 μA (DHA activated current + spadin]. Moreover, spadin was unable to prevent DHA stimulation of mTREK-1 currents; 3.0 ± 0.3 μA versus 3.3 ± 0.2 μA in the presence and absence respectively of spadin (**Figure 2H**).

### Spadin Does Not Antagonize AA-Activation of mTREK-2 Channels

**Figure 3:**
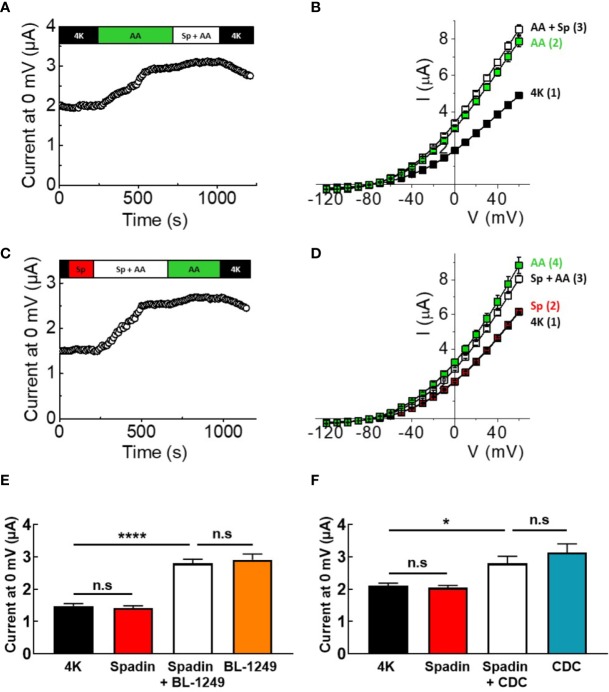
Spadin does not antagonize AA-activation of mTREK-2 channels. **(A, C)** Exemplar time-courses of mTREK-2 currents measured at 0 mV recorded from oocytes by TEVC, under different bath conditions; control 4K (black), 1 μM spadin alone (red), 10 μM AA alone (green) or 1 μM spadin plus 10 μM AA (white) as indicated on bar. **(B, D)** Mean current-voltage relationships of experiments from A and C respectively (*n* = 7-9). Numbers in brackets refer to the order of application of compounds, mirroring respective time-courses. **(E, F)** Mean bar graphs showing the effect of spadin on mTREK-2 current activation by **(E)** BL-1249 **(F)** CDC. mTREK-2 current amplitudes were recorded at 0 mV by TEVC in control bath conditions (4K, black), and following pre-treatment with 1 μM spadin alone (red), 1 μM spadin plus indicated activator (white) and activators alone; 3 μM BL-1249 (orange) or 10 μM CDC (blue). Data are presented as mean ± SEM, *n* = 8-10. *n.s*, not significant (*p* > 0.05), *p < 0.05, ****p < 0.0001, one-way ANOVA followed by Tukey's multiple comparisons test.

Given the effects of spadin on human and mouse TREK-1 channels, and the close biophysical phenotypes of TREK-1 and TREK-2, the effects of spadin were tested on mTREK-2 channels expressed in *X. laevis* oocytes, and assayed using TEVC. **Figure 3A** shows that 1 μM spadin was unable to block mTREK-2 channels pre-activated by 10 μM AA; currents measured at 0 mV were 3.1 ± 0.1 μA and 3.3 ± 0.1 μA in the absence or presence of spadin, respectively (**Figure 3B**). Comparable to observations with TREK-1, spadin alone failed to inhibit basal mTREK-2 currents; 2.2 ± 0.1 μA (control) versus 2.3 ± 0.1 μA (with spadin) (**Figures 3C, D**). Furthermore, the antagonistic effect of spadin observed on AA-activation in TREK-1 channels was absent in mTREK-2 channels (**Figure 3C**). Pre-application of 1 μM spadin showed a small but insignificant antagonization of AA activation of mTREK-2 at 0 mV (**Figure 3D**).

Comparable to mTREK-1, spadin was unable to prevent BL-1249 (3 μM) stimulation of mTREK-2 currents at 0 mV (**Figure 3E**, 2.8 ± 0.1 μA versus 2.9 ± 0.2 μA in the presence and absence respectively of spadin), or CDC (10 μM) stimulation of mTREK-1 currents at 0 mV (**Figure 3F**, 2.8 ± 0.2 μA versus 3.1 ± 0.3 μA in the presence and absence respectively of spadin).

### Ba^2+^ Blocks AA and BL-1249 Activated mTREK-1 and mTREK-2 Channels

**Figure 4:**
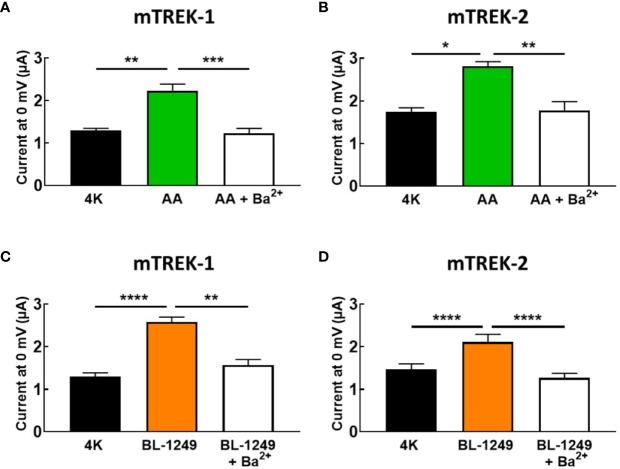
Ba^2+^ blocks AA and BL-1249 activated of mTREK-1 and mTREK-2 channels. Bar graphs displaying the effects of barium ion (Ba^2+^) block of **(A)** AA-activated mTREK-1, **(B)** AA-activated mTREK-2, **(C)** BL-1249 activated mTREK-1, and **(D)** BL-1249 activated mTREK-2 channel currents. mTREK-1 and mTREK-2 channel currents were recorded from *Xenopus* laevis oocytes by TEVC. **(A, C)** Mean currents for mTREK-1 at 0 mV showing the effects of 1 mM Ba^2+^ after pre-activation by 10 μM AA (**A**, *n* = 8) or 1 μM BL-1249 (**C**, *n* = 10). **(B, D)** Mean currents for mTREK-2 at 0 mV showing the effects of 1 mM Ba^2+^ after pre-activation with 10 μM AA (**B**, *n* = 6) or 3 μM BL-1249 (**D**, *n* = 9) activation. Data are presented as mean ± SEM. *p < 0.05, **p <0.01, ***p < 0.001, ****P < 0.0001, Student's paired *t*-test.

Given the absence of spadin inhibition on AA-activated TREK-1 currents, we tested the ability of the classical potassium channel pore blocker, barium (Ba^2+^), to inhibit pre-activated mTREK-1 and mTREK-2 currents. As expected, AA-activated mTREK-1 currents measured at 0 mV showed significant block by 1 mM Ba^2+^ by 46.2 ± 5.3% (2.2 ± 0.1 μA versus 1.23 ± 0.1 μA, **Figure 4A**). Likewise, BL-1249 pre-activated mTREK-1 currents were also inhibited by 39.1 ± 3.0% (2.6 ± 0.1 μA versus 1.6 ± 0.1 μA, **Figure 4C**). This degree of inhibition by barium is close to the 50% block of TREK-1 currents observed in a previous study with 1 mM Ba^2+^ (Ma et al., 2011). A similar degree of block was also observed at 0 mV in mTREK-2 channels pre-activated with either AA (37.2 ± 5.5%; 2.8 ± 0.1 μA versus 1.8 ± 0.1 μA, **Figure 4B**) or BL-1249 (35.5 ± 4.2%; 2.1 ± 0.2 μA versus 1.3 ± 0.1 μA, **Figure 4D**).

### Spadin Does Not Block Structurally Activated mTREK-1 Channels

**Figure 5:**
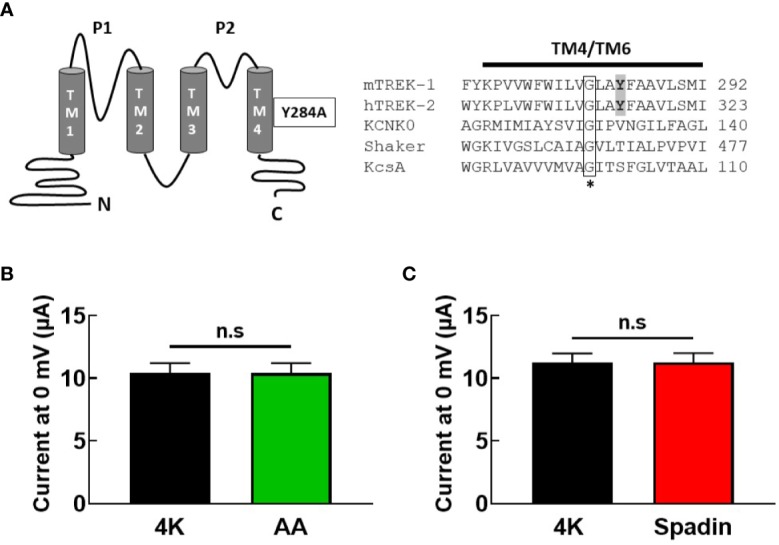
Gating mutation in TM4 reveals spadin is unable to inhibit hyper-active mTREK-1 channels. **(A)** *left panel*, structural diagram of mTREK-1 showing position of Y284A gating mutation, *right panel*, sequence alignment of distal pore-lining transmembrane domains (TM4) of mTREK-1 (NP_034737), hTREK-2 (NP_612191), KCNK0 (AAC69250), (TM6) *Shaker* (CAA29917), and (TM2) KcsA (POA333.1). Highlighted in bold are the tyrosine residue of mTREK-1 (Y284) and the analogous tyrosine in hTREK-2 (Y315). Mutation of Y315 to alanine significantly reduces norfluoxetine activity in human TREK-2 (Mcclenaghan et al., 2016). * Conserved glycine hinge residue (boxed). Mutation to this residue is known to promote stabilization of the open state across the potassium channels listed. **(B, C)** Mean bar graphs of chimera channel currents recorded at 0 mV by TEVC in control bath conditions (4K, black), and following treatment with **(B)** 10 uM AA (*n* = 9, green) or **(C)** 1 μM spadin (*n* = 7, red). Data are presented as mean ± SEM. *p >*0.05, not significant (*n.s*).

Taken together, these findings led us to posit that spadin may antagonize the activation of TREK-1 channels by AA by interfering with the postulated gating transition from the “down” to the “up” state of the TREK-1 filter, *via* a mechanism similar to that proposed for the action of the blocker norfluoxetine on the TREK-2 channel (Dong et al., 2015). We therefore introduced a mutation into the mTREK-1 channel (mTREK-1-Y284A) analogous to the mutation previously described in TREK-2 (**Figure 5A**) which had been demonstrated to significantly reduce norfluoxetine block (Mcclenaghan et al., 2016). This mutation promotes a conformation of pore-lining helices in the “up” state, creating a hyper-active channel. If spadin blocks activated TREK-1 channels as previously suggested, this mutant channel might be expected to be spadin-sensitive.

As anticipated, current magnitude of mTREK-1-Y284A mutant channels was enormous (~10 μA at 0 mV), even when cRNA was serially diluted before injection. Furthermore, mutant channels were no longer sensitive to AA activation; 10.4 ± 2.3 μA (control) versus 10.4 ± 2.4 μA (AA) (**Figure 5B**), indicating a high baseline open probability therefore rendering the channels insensitive to further activation by AA. This prevented the assessment of antagonistic activity of spadin in the mutant channel. However, application of 1 μM spadin alone failed to inhibit mTREK-1-Y284A basal currents; 11.3 ± 1.9 μA (control) versus 11.3 ± 2.0 μA (spadin) (**Figure 5C**), and reinforces our suggestion that spadin is not a channel blocker of activated channels, but selectively and specifically antagonizes AA-activation of TREK-1 channels *via* an allosteric mechanism.

## Discussion

TREK-1 is a background potassium channel, the protagonist in multiple key physiological functions and pathophysiological conditions in nervous and cardiovascular tissue including neuroprotection, depression, epilepsy, pain, ischemia, and atrial fibrillation (Djillani et al., 2019). We have previously shown pharmacological evidence that implicates TREK-1 as a major potassium conductance regulating gastrointestinal smooth muscle contractility (Ma et al., 2018), providing a potential therapeutic target for motility disorders. Although a plethora of physiochemical activators of TREK channels have been characterized, including acidic pH_i_, mechanical stretch, lipids, and compounds including caffeic acid derivatives and fenamates (Vivier et al., 2016), there is a surprising paucity of TREK-1 channel blockers that have the potential to be exploited for therapeutic purposes. Recently spadin, an engineered fragment of a natural propeptide released into blood after the cleavage of prosortilin by furin, was identified as a potent and selective blocker of TREK-1 channels (Mazella et al., 2010). Spadin activity was shown to require pre-activation of the TREK-1 channel by AA, indicating state-dependent binding. Here, using murine forms of TREK-1 and TREK-2, we provide evidence that spadin is not a direct blocker of TREK-1 when expressed in heterologous systems but an antagonist, displaying no intrinsic activity, able to selectively prevent AA-activation of TREK-1 channels through an allosteric mechanism.

Given the inconsistency of our findings with previous studies, critical comparisons were sought. Spadin, a partial NTSR3/sortilin propeptide, has been shown biochemically to directly bind to the TREK-1 channel (note in the absence of AA) and with much higher affinity to the NT receptor (8 nM) (Mazella et al., 2010). Spadin has been proposed to induce a mixture of direct channel blocking effects; inducing a voltage-independent reduction in current (after activation with AA) with an IC_50_ of ~60–70 nM, and channel protein internalization (Mazella et al., 2010). The authors further reveal that the NT receptor (NTSR3/sortilin) itself physically associates with the TREK-1 channel regulating its expression and function. Therefore in those studies utilizing expression systems (COS-7 cells) or primary tissue known to endogenously express the NT receptor (NTSR3, gp95/sortilin), it is not clear whether the action of spadin occurs directly *via* the channel itself or indirectly through the NT receptor. Our data using an embryonic heterologous expression system agree that spadin likely binds directly to the TREK-1 channel in the absence of AA, independent of its activity through the NT receptor. When observed in isolation, spadin clearly works as an antagonist and not as a direct channel blocker. Furthermore, what is not clear from previous studies is how, given its proposed direct block of TREK-1 channels, spadin specifically affects depression with little impact on pain, epileptogenesis or cardiac phenotypes, raising questions about its mechanism of action. However, an antagonistic mechanism would help explain these findings; no intrinsic activity, but preventing activation of the TREK-1 channel in those cells and tissues undergoing specific disease processes.

Our findings agree with those described previously; spadin has no effect on basal TREK-1 channel currents (Mazella et al., 2010) and is specific to TREK-1 (Moha Ou Maati et al., 2012). We show that spadin is unable to inhibit TREK-1 channels activated both chemically (via AA, BL-1249, or CDC), or by *via* structural mutation. Spadin specificity has been argued based on lack of effect in a TREK-1 knockout (kcnk2^−/−^ mice) (Mazella et al., 2010). Mechanistically, if spadin induces effects indirectly through the NT receptor, then removing the coupled effector protein (TREK-1) would consequently remove the response, and does not verify a direct channel blocking effect. Furthermore, it has been suggested that spadin induces internalization of the TREK-1 channel through its action on NT receptors (Mazella et al., 2010), a mechanism that is likely responsible for the long-term clinical impact of spadin seen *in vivo*. However, given that ion channel protein turnover at the membrane occurs on a timescale of hours (Jugloff et al., 2000), this does not explain the acute effects shown in acute electrophysiological recordings.

### Site of Action

We have provided evidence that spadin is specific to TREK-1 and does not antagonize the closely related TREK-2 channel, in line with previous reports (Moha Ou Maati et al., 2012). However, uniquely, spadin antagonism was selective for AA activation, and had no influence on TREK-1 channel activation by other chemical activators, BL-1249 or CDC, or the related PUFA, DHA. A charge cluster in the C-terminal domain of TREK-1 and TREK-2 is considered a crucial site for the activity of bioactive lipid molecules such as AA and DHA (Patel et al., 1998; Kim et al., 2001b; Honore et al., 2002). Given that spadin is a water-soluble peptide, we ruled out an orthosteric mechanism of antagonism involving the C-terminal domain of TREK-1. However that spadin antagonizes AA activation of TREK-1 channels, and not that of DHA, suggests that AA activation of TREK-1 may involve a mechanism distinct from the C-terminal domain, interacting with other regions in TREK-1 or inducing its effects through interactions with the lipid bilayer (Meves, 2008). The related K_2P_ channel, TRAAK, similarly contains a cluster of charged amino acids in its C-terminal domain, replacement of which leaves AA sensitivity intact (Kim et al., 2001a).

Spadin did not antagonize the effects of BL-1249 or CDC, ruling out a mechanism at these binding sites. Schewe *et al*, have recently identified the binding site of BL-1249 (and likely CDC), a negatively charged activator (NCA), to be below the selectivity filter, a conserved site now thought to convey polypharmacological properties to chemical activators across different K^+^ channel families (Schewe et al., 2019). This also suggests that the mechanism of activation of TREK-1 channels by AA and NCA's are different, as spadin selectively antagonizes AA activation, but not BL-1249 or CDC. Spadin could possibly share a similar binding site as the water-soluble TREK channel blocker fluoxetine, and its active metabolite, norfluoxetine. Molecular modeling of TREK-2 in complex with norfluoxetine indicates the inhibitor binds in a pocket underneath the P2 helix of the selectivity filter at a site that is framed by the M2, M3, and M4 transmembrane helices (Dong et al., 2015). However, there is currently no co-crystal structure or molecular modeling simulations of TREK-1 in complex with spadin.

### Mechanism of Action—Allosteric Antagonism

Our data infers that spadin is able to antagonize AA-activation of TREK-1 channels *via* a mechanism that is distinct from the likely site of AA activity, the C-terminal domain, therefore suggesting an allosteric mechanism of antagonism. We hypothesize that the molecular basis of spadin antagonism may be similar to the state-depending binding of fluoxetine in TREK channels. Crystal structures of TREK-1 (Lolicato et al., 2017), TREK-2 (Dong et al., 2015), and TRAAK (Brohawn et al., 2012) channels in the “up” and “down” conformations have been reported, and suggest different conformational gating states. The crystal structures of the hTREK-2 channel in complex with the inhibitor norfluoxetine suggests that conformational changes induced by drug binding can modulate channel activity (Dong et al., 2015). Movement of the pore-lining helices convert the channel between “down” and “up” functional states. The “down” state represents a closed and poorly conductive state stabilized by inhibitors such as norfluoxetine binding within the fenestrations, whereas the “up” state can be induced by membrane stretch or AA activation and represents an open and more conductive state (Dong et al., 2015). Norfluoxetine accesses its binding site only when the TREK-2 channel is in the “down” state. TREK-2 shifts to the “up” functional state when activated by AA, and becomes resistant to norfluoxetine (Mcclenaghan et al., 2016). We posit that spadin works in a similar way; spadin is only able to bind to TREK-1 when the channel is in the “down” state. Binding of spadin restricts the ability of the channel protein to adopt the “up” state conformation upon AA activation. Conversely, spadin is unable to bind when the channel is in the “up” state after pre-activation by AA, and therefore displays no channel blocking effect.

The inhibitory power of norfluoxetine can been markedly reduced by the mutation hTREK-2-Y315A (shown in **Figure 5B**), which promotes a conformation of pore-lining helices in the “up” state (Mcclenaghan et al., 2016). Indeed, mutation of the analogous site in TREK-1, Y284A, produces a channel with all the hallmarks of a channel trapped in the “up” state; very large steady-state current amplitudes and insensitivity to activation by AA. Previous studies suggested that spadin is only able to inhibit channels in the activated state (Mazella et al., 2010), however we observed no inhibition of current in the constitutively active mTREK-1-Y284A mutant.

### Limitations of the Study

The experimental data presented were derived from the *X. laevis* oocyte expression system. Although a well-established and suitable model system for evaluating the biophysical and pharmacological properties of ion channels, there is the possibility that findings of this nature could be inconsistent between different expression systems and cell types. Furthermore, inconsistencies may arise from different methods of peptide synthesis.

Taken together these data demonstrated that spadin displays direct, selective antagonism of AA-activation of TREK-1 channels. Pharmacological and mutational analysis suggests spadin binds to TREK-1 in the “down” conformation at a site distinct from the C-terminal domain, and likely on the extracellular face of the channel protein. Understanding the molecular nature of spadin antagonism should assist in the development of future antidepressant compounds, providing a platform for the design of TREK-1 specific drugs that also lack intrinsic activity. However, further studies involving molecular modeling or structural analysis through co-crystallization techniques are required to determine the exact binding site of spadin on the TREK-1 channel protein, and help further dissect the underlying molecular mechanism of action of this clinically relevant antidepressant.

## Data Availability Statement

The datasets generated for this study are available on request to the corresponding author.

## Author Contributions

Conceived and designed the experiments: AL, RM. Performed the experiments: RM. Analyzed data and wrote the paper: RM, AL. All authors reviewed and approved the manuscript.

## Funding

This work was supported by a Royal Society Research Grant (AL), and in part by the Biotechnology and Biological Sciences Research Council (BBSRC), UK, Grant Number BB/J006114/1 (AL).

## Conflict of Interest

The authors declare that the research was conducted in the absence of any commercial or financial relationships that could be construed as a potential conflict of interest.

## Abbreviations

4-AP, 4-aminopyridine; AA, arachidonic acid; CDC, cinnamyl-3,4-dihydroxy-α-cyanocinnamate; DHA, cis-4,7,10,13,16,19-Docosahexaenoic acid; GI, gastrointestinal; FFA, flufenamic acid; TEA, tetraethylammonium.
