# Supplementary material for: Spadin Selectively Antagonizes Arachidonic Acid Activation of TREK-1 Channels
Source: Front Pharmacol. 2020 Apr 7;11:434. doi: 10.3389/fphar.2020.00434 (PMC7154116; doi:10.3389/fphar.2020.00434)
Supplement: Supplementary file 1 [file Image_1.pdf]

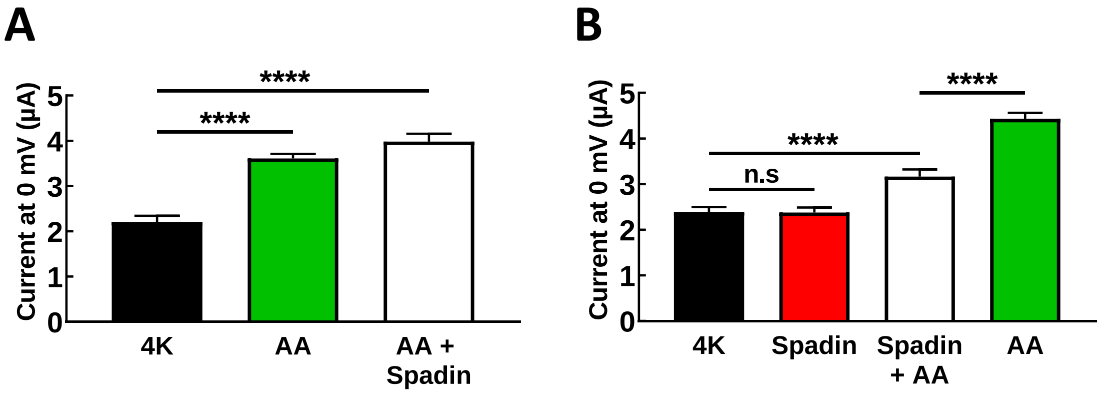

Supplementary Figure 1. Spadin antagonises AA-activation of human TREK-1 channels. Mean bar graphs showing the effect of spadin on mTREK-1 currents amplitudes recorded at 0 mV by TEVC. (**A**) Mean mTREK-1 currents were recorded in control bath conditions (4K, black), and following pre-activation with 10 μM AA (green) and then supplementation with 1 μM spadin (white). (**B**) Mean mTREK-1 currents were recorded in control bath conditions (4K, black), and following pre-treatment with 1 μM spadin alone (red), supplementation with 10 μM AA (white) or 10 uM AA alone (green). Data are presented as mean ± SEM, n = 8-10 for both. *n.s*, not significant ($p > 0.05$), ****$p < 0.0001$, one-way ANOVA followed by Tukey's multiple comparisons test.
